# Palliative Care for People Living With Heart Disease—Does Sex Make a Difference?

**DOI:** 10.3389/fcvm.2021.629752

**Published:** 2021-02-05

**Authors:** Piotr Z. Sobanski, Malgorzata Krajnik, Sarah J. Goodlin

**Affiliations:** ^1^Palliative Care Unit and Competence Center, Department of Internal Medicine, Spital Schwyz, Schwyz, Switzerland; ^2^Department of Palliative Care, Collegium Medicum in Bydgoszcz, Nicolaus Copernicus University in Torun, Bydgoszcz, Poland; ^3^Geriatrics and Palliative Medicine, Veterans Affairs Portland Health Care System, Department of Medicine, Oregon Health and Sciences University, Patient-Centered Education and Research, Portland, OR, United States

**Keywords:** palliative care, symptom control and palliative care, sex related differences, heart disease, breathlessness, spiritual care, holistic care

## Abstract

The distribution of individual heart disease differs among women and men and, parallel to this, among particular age groups. Women are usually affected by cardiovascular disease at an older age than men, and as the prevalence of comorbidities (like diabetes or chronic pain syndromes) grows with age, women suffer from a higher number of symptoms (such as pain and breathlessness) than men. Women live longer, and after a husband or partner's death, they suffer from a stronger sense of loneliness, are more dependent on institutionalized care and have more unaddressed needs than men. Heart failure (HF) is a common end-stage pathway of many cardiovascular diseases and causes substantial symptom burden and suffering despite optimal cardiologic treatment. Modern, personalized medicine makes every effort, including close cooperation between disciplines, to alleviate them as efficiently as possible. Palliative Care (PC) interventions include symptom management, psychosocial and spiritual support. In complex situations they are provided by a specialized multiprofessional team, but usually the application of PC principles by the healthcare team responsible for the person is sufficient. PC should be involved in usual care to improve the quality of life of patients and their relatives as soon as appropriate needs emerge. Even at less advanced stages of disease, PC is an additional layer of support added to disease modifying management, not only at the end-of-life. The relatively scarce data suggest sex-specific differences in symptom pathophysiology, distribution and the requisite management needed for their successful alleviation. This paper summarizes the sex-related differences in PC needs and in the wide range of interventions (from medical treatment to spiritual support) that can be considered to optimally address them.

## Introduction

Heart failure (HF) is a global epidemic, having a complex epidemiology and an estimated prevalence of almost 38 million individuals globally (1, 2). It is a common end-pathway of many cardiovascular diseases. HF causes a substantial burden for the numerous individuals affected and their relatives, even under optimal cardiological care, it is also the leading cause of mortality in many populations. As HF is a polyetiological syndrome, differences in the distribution of specific HF types between women and men in different age groups mirror the prevalence of underlying diseases in individual ethnic and geographical populations. The quite universal clinical syndrome evoked by heart dysfunction, especially in advanced stages, consists of breathlessness, exercise intolerance, tendency to hypervolemia and tissue hypoperfusion in the end stages. This can be complicated by features of individual underlying disease (like angina in people with HF of ischemic etiology, neuropathy in those affected by the wild type of amyloidosis, or hemoptysis in the case of pulmonary arterial hypertension), age related problems (i.e., frailty syndrome or dementia) and/or concomitant disease (i.e., degenerative arthrosis, peripheral vascular disease, diabetic neuropathy) (3). Two other symptoms commonly seen in people affected by HF are depression and fatigue. They often coexist, with a complex etiology and influence the perception of other symptoms such as pain or breathlessness (4). The majority of people living with HF experience daily a number of symptoms limiting their functioning, quality of life (QoL) and negatively affecting their life-expectancy (5). Optimal cardiological care could be improved by the concomitant provision of palliative care (PC).

## Definition of Palliative Care

PC has evolved in recent decades to become a discipline caring for people living with serious diseases whose' health status does not respond fully to the disease specific treatment. They typically have health related symptoms, problems and needs that can, if complex, be addressed by a multidisciplinary team (consisting at least of medical and nursing staff, psychologists, social workers, physiotherapist, occupational therapists, chaplains) with the goal of improving QoL, even whilst the underlying disease is progressing or entering the terminal phase (6). Unfortunately PC is misconceived as being synonymous with end of life or hospice care and falsely understood as an approach dedicated to those dying from cancer. In fact, if the recommended care pattern is implemented in a timely fashion alongside specialist (e.g., cardiological) care, it can benefit many people living with advanced diseases, including HF, by decreasing the burden caused by the symptoms (such as pain, breathlessness, fatigue, depression), improving QoL and spiritual well-being (6–14). Studies investigating the influence of PC in a population affected with HF are scarce and show a modest improvement in QoL when PC has been added to standard cardiologic care (9, 11, 15, 16). The sex related differences on the efficacy of PC interventions for people living with HF has been investigated by only one single center study. The study population of just 150 people (71 women, and 79 men) was randomized in a 1: 1 proportion to usual care and usual care plus PC. Improvement in the QoL scores with PC interventions have been proven with men only, despite women experiencing a greater symptom burden (17). Just changing the perception of PC from a discipline providing end of life care to one focused on improving QoL could be enough to improve access to PC (18). PC added to optimal cardiac care, rather than replacing it, is still underused despite being recommended by both palliative and cardiological societies. A recent analysis of a large US database with a national in-patient sample has shown that from 2002 to 2017 on average only 4.1% of people who had been discharged after acute HF hospitalization had a PC encounter. There has been, however, some improvement over the last 15 years (from 0.4% in 2002 to 6.2% in 2017), but even recently only 6.5% of women and 5.9% of men encounter PC, predominantly when they are suffering from a terminal condition (19). The median time from first specialist PC consultation to death between 2006 and 2011 was only 21 days in a single center study (20). Qualitative/narrative study showed that HF patients and their relatives who received PC concurrently to cardiac care, whilst being in III or IV NYHA (New York Heart Association) class, wished they had received PC interventions earlier in their care, particularly at the time of diagnosis of advanced HF. In contrast, the clinicians representing primary care and cardiology interviewed reported concerns about the overly early implementation of concurrent PC (21).

The management of both physical and psychological symptoms, support in decision-making, coordinating care, social assistance, and spiritual support all are elements of PC. To make optimal medical decisions, the integration of the patients' personal values with their knowledge and understanding about disease progression and the possibility of both improvement and deterioration/death should be ensured. Such an individual approach, based on sensitive in-depth communication, could support or prevent invasive interventions and hospitalizations. Some interventions may not correspond with personal values and wishes, or be perceived as too burdensome (22). Such advance care planning reduces readmissions and costs and increases the satisfaction with the care received (16, 23). The involvement of PC should be triggered by needs rather than the risk of deterioration or death. Unfortunately, the second pattern still dominates, postponing PC provision to the moment of active dying or even preventing it completely (6, 8). PC can be provided in the form of primary (called generic) PC to most people living with HF by health care professionals with a knowledge of PC principles, or in form of specialist PC delivered by clinicians with special training, possessing knowledge, skills, and competencies to address difficult to treat symptoms, existential distress or more complicated problems (24).

## The Epidemiologic Difference Between Women and Men Affected by HF

Men prone to macrovascular coronary artery disease and myocardial infarction are at almost twice the risk of HF with reduced ejection fraction (HFrEF) and are usually younger at the time they are affected by HF than women. Women are more susceptible to microvascular dysfunction/endothelial inflammation, thus are at higher risk of HF with preserved ejection fraction (HFpEF) and are usually older at the time of diagnosis (25–27). Among people affected by HFpEF, men suffer from greater limitations in terms of functional capacity, have more comorbidities and higher cardiac mortality (death caused by refractory HF and sudden cardiac death); women die more often of infections and cancer, but the all-cause mortality is similar between both genders (28). Post-partum cardiomyopathy only affects women up to 6 months after delivery and Takotsubo cardiomyopathy or pulmonary arterial hypertension predominantly affects women (the ratio between women and men is 9:1 for Takotsubo cardiomyopathy) (26, 27). Women with advanced HF are older than men, they are less likely to be married or to be in a domestic partnership, more often widowed, and are more likely to be dependent on institutional support (17, 27, 29).

## Quality of Life of People Living With Heart Failure

There are many concepts concerning the definition and components of QoL and numerous instruments for assessing it. Some tool, like the disease-specific Minnesota Living with Heart Failure questionnaire, or generic ones such as the Medical Outcomes Study SF-36—used commonly to assess QoL of people living with HF—focus on the negative impact of health on pre-specified items and thus reflect disease advancement rather than patients' self-reported QoL (30–32). Such instruments for assessing symptoms/disease related limitations in daily living and distress used for measuring health-related QoL (HRQoL), show the constant deterioration of QoL in parallel to disease status (33). Over 80% of people living with HF report physical symptoms such as dyspnoea, fatigue, oedema, sleeping difficulties, and chest pain, all negatively impacting QoL (33, 34). Emotional status and depression can significantly diminish QoL, exaggerate the experiencing of symptom burden, and be aggravated by physical symptoms (35).

QoL is, however, more complex than described above and reflects the multidimensional impact of a clinical condition and its treatment on a person's daily life. It is a subjective experience encompassing emotional status, social functioning, and symptom burden and merely reflects their objective clinical, or physiological status. In other words, QoL can be defined in a more comprehensive way as the ability to maintain happiness, engage in fulfilling relationships and perform physical and social activities. Many people living with even advanced HF can perceive their QoL as good, despite suffering from symptoms and experiencing limitations in physical and social functioning (31).

PC goes beyond limiting symptom burden and addresses more comprehensive dimensions of human life including psychosocial, existential, spiritual problems as well as providing support for family and informal carers. There are gender related differences in QoL in people suffering from HFrEF: women with HFrEF have worse HRQoL compared to men assessed by the Kansas City Cardiomyopathy Questionnaire (KCCQ, a disease-specific instrument); and EuroQoL 5 dimensions (EQ-5D, a generic instrument). Women report higher symptom frequency, symptom burden, physical limitations and social limitations, as well as lower QoL. These differences do not appear to be mediated by clinical or biological factors (such as age, body mass index, systolic blood pressure) classically associated with HRQoL nor with HF severity (17, 27, 36–38).

## Symptom Burden

Symptoms affecting people living with advanced HF surprisingly do not differ substantially from symptoms reported by people living with advanced cancer who receive PC (39–41). There are only a few significant differences in patients with HF: they suffer more often from dyspnea that is higher in intensity, report reduced appetite almost as frequently, albeit less intensely, and have almost as much pain but which is slightly less severe in comparison to patients with advanced cancer (40). Women experience a greater symptom burden and suffer more frequently from depression than men, despite similar or even less advanced HF (17, 27, 37). Using a comprehensive and reliable questionnaire (i.e., Memorial Symptom Assessment Scale for HF—MSAS-HF), people living with HF report about 13.6 symptoms on average, despite optimal medical management of HF (42, 43). Each of those symptoms should not be considered individually. Some symptoms are seen in clusters (breathlessness, anxiety, and depression termed a distress cluster; fatigue, drowsiness, nausea, and reduced appetite—referred to as a decondition cluster; pain, and a sense of generalized discomfort—known as a discomfort cluster), with relatively small to moderate correlations between clusters, suggesting the existence of a common pathway or interdependence for symptoms grouped in one cluster (44).

Symptom burden and distribution differ between females and males. Women affected by HF report a higher symptom burden for pain other than chest pain, dry mouth, swelling of the arms and legs, sweats, feeling nervous, fatigue, nausea and vomiting (43, 45). Men suffering from HF report a higher burden with sexual problems (they were, however, more often married than women, which might clarify why they were more likely to report this issue as a problem) (43). A review of patient records indicates that there are substantial differences in how health care professionals perceive symptom burden in women and men—females had to report a higher level of distress than males in order to get their symptoms acknowledged, documented and managed (46, 47).

Some studies have shown an association between depression, fatigue, pain, and breathlessness (4, 48–50). The relationship between depression and physical symptoms is bidirectional—people suffering from depression perceive more intense physical symptoms and conversely people affected by physical symptoms are more prone to suffer for depression (51–53). The top-down (predictions, anticipation, modulation) and bottom-up (afferent signaling) theory, stress the role of the integration of both centrally and peripherally originating signaling in processes of stimulus initiation, transmission and processing in symptom perception. This clarifies the crosstalk between emotional status, memory, and meaning with the sensitivity of peripheral receptors (54).

Symptoms in people living with HF do not correlate with objective measures such as left ventricular ejection fraction (LVEF), right heart catheterisation parameters, serum creatinine, hemoglobin, amino-terminal pro-B type natriuretic peptide (NT-proBNP) concentrations and only poorly with peak oxygen uptake (55–60). However, one study reports the severity of HF symptoms relates to decreased ventricular compliance in women, but not in men and to the dilatation of the left ventricle, but only in men. Larger left ventricle size is associated with better physical symptoms for women and worse physical symptoms for men (60). All this suggests that there is no simple link between the degree of heart or circulation system dysfunction and symptoms.

## The Elements of PC Interventions

A fundamental for PC is symptom management (61). Patients living with serious disease, including those with HF, identify symptom management as a top priority, particularly at the end of life (62). Despite this, only a minority of people living with advanced HF receive management and care focused on symptom alleviation (62). The three most common symptoms affecting people living with HF are pain (prevalence over 80%) shortness of breath (prevalence 65–75%) and a lack of energy (79–76%) (42, 43, 63). The last two symptoms are perceived as a hallmark of HF and are commonly used for the classification of HF advancement (according to NYHA) but unfortunately, they do not trigger interventions aimed at alleviating them, even if they are severe. The gap between frequency of documented symptoms and interventions prescribed to alleviate them can be as high as 60% (64). The upper mentioned three most frequent symptoms (non-cardiac pain, breathlessness and lack of energy) are also the most severe and most distressing symptoms (43).

### Symptom Management in People Living With HF

#### Pain

Pain, the most commonly reported symptom by those living with advanced HF, affects up to 84% of those affected by heart failure (29, 65). Its frequency increases along with the severity of HF (up to 89% of people in IV HYHA class) (63). Pain, other than chest pain (reported as well, as non-cardiac pain), predominates and affects up to 77% of people living with HF (43). It is only rarely perceived by health care professionals and identified as a target to address. That is why it is underreported and undertreated (39, 40, 50, 63, 66). Pain is not only one of the factors limiting QoL, but it also negatively influences HF pathophysiology (66). Uncontrolled pain stimulates the sympathetic nervous system and activates the renin-angiotensin-aldosterone system, all of which lead to increases in the haemodynamic workload, sodium and water retention and finally to HF decompensation and a higher risk of rehospitalisation (66, 67). Untreated pain additionally increases the use of non-steroidal anti-inflammatory pain killers (NSAID), including those contraindicated in HF, worsens self-monitoring and self-management (risk factor of HF decompensation and hospitalization) (66, 67) and increases the risk of depression (a factor limiting QoL and increasing the risk of HF related hospitalization and mortality in people with HF) (48, 68–70).

Successful and safe pain management in people living with HF is more challenging than in people without HF, but can decrease mortality in people with cardiovascular disease (71–73). The best-known framework for treating pain is known as the WHO analgesic ladder. It aids in decision making over the choice of painkillers. Non-opioids are recommended for mild pain (step I), weak opioids for moderate pain (step II) and strong opioids for severe pain (step III), always with the addition of adjuvants, if appropriate. Unfortunately, most non-opioids, particularly NSAIDs carry the risk of worsening HF, renal function and atherothrombotic events, and are contraindicated in people with cardiovascular disease, including HF (10, 74–77). Two non-opioid pain killers seem to be free of those side effects, namely paracetamol and metamizole. Both lack an anti-inflammatory effect and cause other potentially serious side effects (hepatotoxicity and bone marrow suppression, respectively). Weak opioids (step II of the WHO analgesic ladder), tramadol and codeine, are prescribed with decreasing frequency, due to their variable pharmacokinetics and the risk of tachycardia and hyponatremia, and tramadol additionally due to risk of orthostatic hypotension and falls in people over 65 years (78, 79). Strong opioids (step III of the WHO analgesic ladder) are recommended for treatment of moderate to severe pain. A small dose of strong opioids (up to 30 mg of morphine or 20 mg of oxycodone) has recently been proposed as step II on the analgesic ladder (80). [For details on treating pain in people living with HF, see the recent EAPC expert position statement (6)].

For those reasons, the most commonly recommended measures to treat pain in people living with HF are topical interventions, non-pharmacological techniques and prescribing strong opioids (81). The last, especially if not properly used, bear potential serious side effects, including addiction and the risk of opioid related death (82). Surprisingly, there is a lack of evidence demonstrating the superiority of opioids over other analgesics in treating chronic non-cancer pain (83). Two strong opioids, buprenorphine and methadone, may prolong the QTc and thus are not recommended in people with borderline prolongation of QTc (450–500 ms) and are considered as contraindicated if QTc exceeds 500 ms (79). Additionally, the safety of strong opioids in patients with advanced HF has not been extensively studied, but some research suggests that they represent a source of potential harm, specifically to this population. One retrospective study has shown increased risk of ICU admissions, the need for ventilators, prolonged hospitalization and higher mortality in people with acute HF who have been exposed to opioids (84). A cohort study revealed that using opioids was associated with increased risk of coronary heart disease and cardiovascular death among females but not males (85). Opioids might increase the risk of atrial fibrillation—individuals with an opioid prescription develop this arrhythmia 34% more often than those without it (86, 87). Recent studies suggest that morphine increases 4.37-fold the risk of developing AF in women with breast cancer, but this is abolished by antioestrogen treatment with tamoxifen. The risk of AF is especially high in current morphine users of all ages with a low Carlson Comorbidity Index score, and rises along with the duration of morphine use (88). The tamoxifen protective effect may be related to the specific pharmacologic effect of the drug or be an indirect consequence of estrogen deprivation. This is in line with the hypothesized detrimental effects of opioids on cardiovascular risk in women described above.

The prevalence of symptoms, including pain, depends on biological/chromosomal (sex related differences) and socio-cultural (gender related) factors. Studies, if they have even considered the differences in the experiencing of pain between women and men at all, analyzed only biological sex. Socio-cultural factors' impact on symptoms in HF have not yet been investigated. There are social and cultural influences on pain experience in humans, and thus men and women experience pain in a way that conforms to gender expectations. For this reason, gender has an impact on pain reporting—it is socially accepted that women tend to report more pain than men and have a lower tolerance for pain (89). Few studies have explored the role of biological sex as it pertains to the safety of prescribing of opioids in patients with chronic pain. There are several reasons why opioids might be prescribed differently to men and women, including differences in pain perception (90). It is hypothesized that the sex dependent biological factors influence differences in the perception of chronic pain, that they are related to substantial differences in the functioning of the immune system, and that they play a crucial role in chronic pain syndrome. Based predominantly on animal studies, it seems that the immune system (inflammation in the spinal cord around pain transmitting pathways) functions differently in females and males. Females predominantly utilize T-cells while microglia in the spinal cord in males mediates the modification of chronic pain (89). Whether this observation has a clinical implication with respect to different perceptions of pain and the varying degree of effectiveness of pain killers is currently unclear (89). Previous research suggests that women are more likely to be prescribed opioids, but men tend to receive more potent agents (91–94). Long-term opioid use was substantially higher among older women than it was among younger women or men in any age group (93). A cohort study spanning 13 years using the healthcare records of 32,499 individuals aged 15–64 who commenced chronic opioid therapy for non-cancer pain showed that men are at a higher risk than women of escalation to high-dose opioid therapy and death from opioid-related causes (82). This can be a consequence of more attention being paid to pain reporting by men and more intense efforts to alleviate it. Older women have a lower risk of opioid misuse but may be more vulnerable to the adverse medical effects of opioids such as sedation, falls, constipation, respiratory depression, dysphoria, accidental overdose, and medication interactions (95). Women are at a greater risk of undertreatment of pain, although the use of both prescription and non-prescription analgesics is significantly higher among women than men (90).

Despite many doubts regarding the safety of pain management in people living with HF, optimal pain alleviation has to be achieved since uncontrolled pain increases the risk of HF related hospitalization and cardiovascular mortality (66, 73).

#### Breathlessness

Dyspnoea is a hallmark symptom in advanced HF. It is defined as the subjective, multidimensional experience of breathing discomfort (96). Breathlessness, if unrelieved and severe, can be devastating to a person's QoL and is associated with poor survival rates (97). The perception of breathlessness is driven by a mismatch between demand for ventilation (sensed by chemo- and metabo-receptors) and actual ventilation (sensed by pulmonary stretch receptors, pulmonary C-fibers, chest wall joint and skin receptors, and skeletal muscle ergoreceptors) (98). Breathlessness, especially in its chronic form, does not correspond with any sign that can be objectively seen in clinical examination or any parameters that can be tested (such as breath rate, saturation, echocardiographic data, pulmonary wedge pressure or blood tests) (6). The language of breathlessness (how a person describes it) is complex and indicates its complex pathophysiology (99). Breathlessness can vary respective character, intensity, unpleasantness, emotional and behavioral significance. It is classified as acute, chronic (having usual fluctuations with regard to the above-mentioned features) with usually superimposed episodes of exacerbations (they can be triggered, by predictable or unpredictable, factors or non-triggered). Those episodes of breathlessness go beyond the usual fluctuations (100). The most commonly seen triggered, predictable episodes are usually provoked by physical activity, with breathlessness accompanying exercise with gradual onset, sometimes becoming very intense—in healthy people with heavy exertion (perceived as normal breathlessness, mostly not unpleasant), but in people with HF, especially if this is advanced, it is precipitated by moderate or slight exercise (perceived as unpleasant) (101). This kind of breathlessness is a universal feature of HF (even if optimally treated) and relates to the skeletal myopathy that is present in the HF syndrome of any etiology. As HF progresses, the episodes of breathlessness can be seen at rest—typically after taking up a supine position, sometimes with wheezing and coughing (asthma cardiale) or bending forwards (102, 103). Breathlessness is so ubiquitous in people living with HF that it has become the basis for the most commonly used classification of HF according to NYHA (104). Breathlessness, is also common in many other conditions like infectious, lung, renal, metabolic, hematologic, neuro-muscular or even psychiatric disease, and so more than one pathology can often evoke it in one person. Before considering the symptomatic (i.e., palliative) management of breathlessness, its etiology and the possibility of specific treatment have to be actively sought.

In women more often than in men with heart disease, breathlessness can be equivalent to anginal pain (105). In people affected by HF, blocking neurohormonal activation, optimizing afterload, heart rate and volaemia are the principles of breathlessness management. Even in end-stage-disease using vasodilators/neurohormonal antagonists, heart rate controlling interventions and drugs as well as diuretics improves dyspnoea. It has been shown that the continuation of these drugs, sometimes in modified doses, improves the QoL, even in advanced HF (106–108). If the cause of breathlessness cannot be treated specifically, and if the breathlessness is severe or disabling (corresponding with III or IV NYHA class), symptomatic treatment should be considered as mandatory, unfortunately it often remains untreated. Acute breathlessness is perceived as an alarming symptom for both patients and health care professionals. It is 1 of the 10 leading causes of all emergency room visits (5%), 20% of those delivered by ambulance and causes 25% of hospitalisations (109, 110). Chronic breathlessness, affecting the everyday lives of almost 9% of the general population, remains “invisible” i.e., unnoticed as indication for symptomatic treatment, even if the people suffering from it are unable to walk more than 100 m or to leave home (111, 112). This invisibility of breathlessness affects health care professionals (as patients examined at rest do not demonstrate breathlessness, even if the exercise threshold for inducing dyspnoea is very low) but surprisingly the patients themselves as well (due to the omnipresence of breathlessness in their life). Finally, given the lack of established, effective standards in breathlessness alleviation, healthcare professionals do not ask about symptoms that they feel unable to alleviate (111). The treatment gap in the case of dyspnoea can be as high as over 70% in hospitalized patients with acute HF (113). 42% of patients hospitalized for decompensated HF report no improvement in dyspnoea 1 week after discharge in comparison to admission (114).

**Figure 1:**
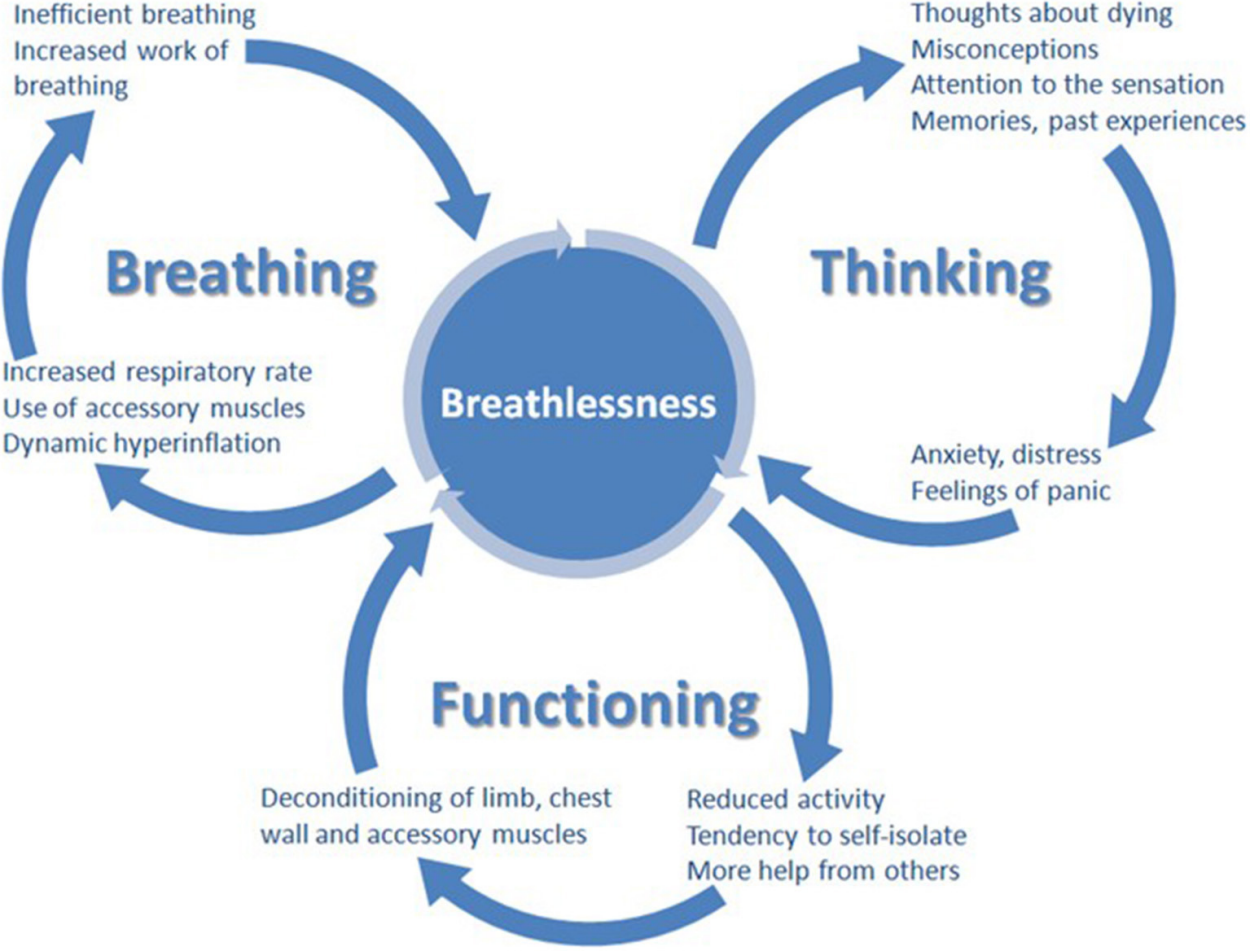
The breathing, thinking, functioning clinical model (98).

Many people living with HF suffer from breathlessness, or its resulting limitation in daily activity, despite optimal cardiologic treatment. Similar conclusions come from studies in people with chronic respiratory disease, in those optimizing the treatment of the underlying disease has an inconsistent impact on the symptoms. All this suggest that even optimal disease specific treatment cannot be only intervention to ameliorate breathlessness; symptomatic interventions are needed (98). Non-pharmacological (physiotherapy, breathing-relaxation training, cognitive, behavioral strategies, walking-aids, hand-hold ventilators) and pharmacological management should be considered (6, 115, 116). There are a plethora of non-pharmacological approaches to ameliorate breathlessness, without evidence to guide the individualization of therapy (98). Multi-modal, non-pharmacological approaches that work concurrently at multiple points within the brain, respiratory and skeletal system offer the most successful amelioration of breathlessness (117–119). Without proper support, people suffering from breathlessness reduce their activity and thus become increasingly deconditioned, in turn worsening breathlessness. This mechanism could in part explain the progression of breathlessness severity, despite the fact that the underlying disease remains stable (98, 120). A recently developed clinical model, the “Breathing Thinking Functioning” (BTF), stresses the importance of the cognitive and behavioral reactions responsible for the worsening perception of dyspnoea in people with chronic obstructive pulmonary disease (Figure 1). Parallel interventions affecting all domains should be provided to improve the alleviation of breathlessness (Table 1). Oxygen can be tried, but improvement is to be expected mainly in hypoxemic patients (121). The basis for pharmacological treatment are low-dose opioids, usually morphine titrated up to 30 mg orally/day in divided, appropriately to formulation, doses (or oxycodone in equivalent doses), but their efficacy and safety in people living with HF is still not well-established [for more details, see the recent EAPC expert position statement (6)]. Some studies even suggest that harm can be caused by using opioids for this indication in people with acute heart failure (84). Benzodiazepines are widely used, but do not improve breathlessness and cause serious side effects, including sedation, increased risk of death, falls and pneumonia, and for those reasons, except for uncommon situations when anxiety plays really a crucial role (usual in case of acute breathlessness, especially with panic attacks), they should be considered as contraindicated (122–126).

**Table 1:** The categorization of symptom management approaches according to breathing, thinking, functioning domain (98).

**Breathing**	**Thinking**	**Functioning**
Breathing techniquesHandheld fanAirway clearance techniquesInspiratory muscle trainingChest wall vibrationNon-invasive ventilation	Cognitive behavioral therapyRelaxation techniquesMindfulnessAcupuncture	Pulmonary rehabilitationActivity promotionWalking aidsPacingNeuromuscular electrical stimulation

Breathlessness affects women more often than men. In the general population, the prevalence of chronic breathlessness is almost twice as frequent in women in comparison to men (odds ratio, OR 1.9, *p* < 0.001) (112). A similar trend has been reported in those affected by HF, however the magnitude of the difference is smaller; for dyspnoea at exertion OR 1.2, *p* < 0.001 and for rest dyspnoea OR 1.19, *p* = 0.01 (25).

#### Depression

Depression is up to four times more frequent in people living with HF (21.5%) than in the general population (2.6 in males and 7% in females) (70). Significant differences in the prevalence of depression exist between those who are hospitalized and outpatients with HF (13–77% vs. 13–48%, in different studies) (68, 70, 127–129). The meta-analysis indicates the prevalence of depression among different groups. Its prevalence rises with HF severity (11% in I NYHA class, 42% in IV NYHA class) and is an important factor limiting QoL, increasing the risk of hospitalisations, emergency room visits and death (48, 68–70). Some studies reported that anxiety, depression and psychological distress are more frequent in females than in males (64 vs. 44%), with 37% of women vs. 24% of men with advanced HF suffering from current depression (17, 27, 47, 69, 130). Patients with higher levels of depression had a higher total symptom burden (43). Based on this observation, it has been hypothesized that the effective management of depression could be one measure to improve the general symptom burden in people living with HF. Intensity of anxiety, depression, and psychological distress seems to be higher in female patients when they are accompanied by decreased social functioning, limits in pursuing hobbies, increased dependency or a disturbed body image. Depression in patients older than 51 years after myocardial is almost twice as frequent in women than in men (15–19% vs. 9–14%) (131). Tricyclic antidepressants are contraindicated in people living with HF, due to their negative inotropic and proarrhythmic properties. Sertraline does not cause an additional risk for this population, and venlafaxine can even reduce the risk of HF in the general geriatric population, so both are considered drugs of choice in HF (132, 133). Selective serotonin inhibitors can precipitate however syndrome of inappropriate antidiuretic hormone secretion and as consequence hyponatremia, especially in older women. For this reason caution is needed and monitoring of natrium in serum, already several days after starting this drugs is required (134).

### Spiritual Care and the Whole Person Care Approach

A mandatory mission of PC in modern medicine is to remind everybody of the potential to find new realistic hopes, to develop his/her creativity and to grow as a person, even in the most difficult situations such as dealing with his/her own imminent dying/death. One of the dimensions of growing significance, especially as a disease is progressing, is a person's spirituality, which explains why spiritual care has to be an integral element of PC (6). Spirituality is the way a person seeks and expresses the meaning and purpose of their own life, and the way they experience their connectedness to the moment, to themselves, to others, to nature and to the significant or sacred and goes far beyond religiosity (135). According to EAPC, spirituality is multidimensional and consists of existential challenges, value based considerations and attitudes and religious considerations and foundations (136). The “whole person care” concept extends the goals for medicine as a whole in the twenty-first century, not only PC. This shifts the focus from just curing (treating a disease) to healing (treating the patient as a person). The process of healing is defined as becoming psychologically and spiritually more integrated and whole, enabling a person to become more completely her- or himself and more fully alive (137). To empower this phenomenon, the recognition of the central place of spirituality in a persons' life and the importance of the relationship between the clinician and patient are needed (135, 138, 139). Thus spiritual care is understood as an integral part of PC and, along with the whole person care approach, has started to be recognized as the optimal model of caring (6).

The evidence shows the positive impact of spirituality on treatment efficacy, prognosis, mortality and better coping of the patient and his/her relation to clinicians. Spiritual peace better predicts the mortality of people with HF than comorbidity and functional status (140). Higher level of religiosity/spirituality or greater spiritual well-being is associated with less depression, (141) lower anxiety (142) and better resilience (143). Quality of religious coping, seeking spiritual support and help from God is associated with less distress among patients undergoing cardiac surgery (144). Spirituality has also been shown to be related to self-management and lifestyle changes in people with heart disease (145). Praying positively affected QoL and the psychological status of patients who have undergone a pacemaker implantation (146) and self-care of elderly patients with HF (147). The provision of spiritual-religious interventions has also led to the improvement of life satisfaction and depression rate among elderly patients with HF (148). The trajectories of social and psychological well-being track the physical decline observed at the end-of-life of people with HF, however spiritual distress reveals independent background fluctuations (149). Spiritual well-being remains stable for up to 30 months during observations among advanced HF patients and is lower for those with more symptom distress (150). However, if a gradual decrease is observed, it may reflect a progressive loss of identity and growing dependence (151). Religious beliefs, love, hope and trust help to increase spiritual well-being even at the very end of life. Importantly, people who felt valued by their clinicians were more able to find a sense of their own worth and meaning (149). Such a healing relation and basic spiritual care begins from the therapeutic presence of the clinician (being on hand, i.e., “here and now”), from the enhancement of the patient's dignity and his or her need to be respected as an unique human being, from asking about spiritual needs of the patient and cooperation with a chaplain and other people involved in spiritual care. EAPC recommends that clinicians caring for people should respectfully inquire about the patients' spiritual needs and, if they wish, make time to address them as they would with physical concerns (6).

Are there sex related differences in the spiritual needs of the patient and modes of spiritual care? Any comparison of spirituality/religiosity among men and women appears to be complicated. Evidence from a meta-analytic sample representing nearly 126,000 participants suggest that the relation between spirituality/religiousness and health differs between men and women and that researchers should separately estimate those two models (152). One partial explanation proposed for this phenomenon was differences in the psychosocial resources that men and women receive from religious involvement, with women being more religious and living longer, thus may have stronger network connections and benefit more from them compared to men when elderly. As an example, both men and women attending services at least once a week (compared with those who attend less frequently or never) have between a 1.1 and 5.1 years longer total life expectancy and between 1.0 and 4.3 years longer activities of daily living, disability-free life expectancy (153). However, these differences in total, disability-free, and disabled live expectancy across religion groups tended to be larger for women than men, which may be partially related to the influence of social support and network integration. Some studies suggest gender related differences in images of God or in the ways of applying religious coping strategies and in the use of positive and negative religious coping (154–156). Another study revealed while men and women suffering from serious or life-limited illness endorsed an overall similar level psycho-social-spiritual healing, women were shown to have greater enjoyment of mind-body practices, including prayers, gratitude, compassion and a desire to be more positive than men (157). Evidence they may experience introspective and reflective processes of healing in a different way may have some practical implications in choosing specific therapeutic interventions. Very few studies explore this topic specifically among people with HF. One of the few is a longitudinal observation of more than 180 elderly people with heart disease assessing whether gender and the existence of cardiac health problems affected older adults' spiritual and religious involvement after 12 months (158). While women in poor cardiac health turned toward prayer and devotion, older men with cardiac problems engaged in more religious doubt and questioning which seemed to be a new coping strategy for them. The study suggests that spiritual interventions directed to help elderly men with heart disease should recognize the likelihood of a patient's religious doubt and existential questioning. Nevertheless, two main conclusions related to the potential sex differences in spiritual care among people with HF can be made: 1/ there is no typical pattern of spiritual needs for men or women, thus spiritual needs assessment and support should always be tailored individually; 2/ spiritual history and screening for spiritual needs should be done for each PC patients, not as a once-only activity, but as a process of caring and developing healing relations. And this is in agreement with the recent EAPC white paper recommendations regarding how one should educate clinicians on spiritual care for patients receiving PC (136).

### Care for Carers

HF is one of most common chronic diseases leading to disability and a need for long-term care. Home based assistance is becoming a mandatory strategy to support and care for those in this condition. In Europe, the number of informal caregivers range from 10 to 25% of the total population, yet they provide 80% of all long-term care.

PC acknowledges caring for unformal carers, their well-being and ability to care for their ill loved ones as one of its tasks. Unrelieved symptoms not only burden patients but their caring relatives as well. A higher severity of breathlessness corelates with worse carer psychological health, indicating not only the need for optimal symptom management but also for support for the informal caregivers, especially in the case of severe dyspnoea (159). The relatively sparse studies on sex related differences in caring suggests that women, including those who are elderly and fragile, provide the majority of family caregiving for older adults. The higher proportion of women is linked to the societal expectation that they should provide care at the end-of-life for family members. They experienced a greater degree of mental and physical strain, higher levels of distress and burden as well as worse QoL than males. Women's psychological distress was associated with the health condition of their partner, whereas men's psychological distress was found to only be associated with their own health condition. Unfortunately, the burden of informal caregivers remains mostly unrecognized and the need for support is usually uncovered. Health care professionals should provide assistance and support more sensitively for older females caring for their relatives (160–162). Many relatives feel burned out from the length of time they have spent being a caregiver (21). These observations suggest that providing institutionalized care at the end of life should be considered even if family care in the community is theoretically possible. The aim of this would be to give support to mostly older women caring for their loved ones to prevent physical and psychosocial burden.

The differences between women and men in relation to PC for people living with HF have been summarized in Table 2.

**Table 2:** Differences between women and men in relation to PC for people living with HF.

**As a person suffering for HF**	**Women compared to men**
Age and concomitant diseases	More likely to be older and to have a history of hypertension and diabetes mellitus.
Characteristics of HF	More common HFpEF
In case of HFrEF	Severity of symptoms depends on lowered ventricular compliance and not on dilatation of LV (inversely in men)
Cause of death	More common non-cardiovascular deaths
**Symptoms**:Pain other than chest pain, dry mouth, swelling of the arms and legs, sweats, feeling nervous, fatigue, nausea and vomiting	Higher symptom related burden
Self-reported breathlessness at rest and with exertion	Significantly higher rate
Comorbid depression and anxiety	Higher rate
Depression treated with medication	Higher prevalence
Pain management	Greater risk of undertreatment of pain in spite of higher use of prescription and non-prescription analgesics
Opioid use	Potentially higher risk for atrial fibrillation related to opioid useLower risk of escalation to high dose and death from opioid-related causesLower risk for opioid misuse (for older women) but more vulnerability to adverse medical effects of opioids
Quality of Life measured by Kansas City Cardiomyopathy Questionnaire (KCCQ)	Significantly lower, despite similar physician assigned NYHA class
Psychosocial needs/aspects	More poorly cope with the diseaseMore likely to be widowed or aloneIncreased reliance on family as caregivers and more likely dependence on institution
Spiritual needs	More often religion deeply important
Impact of PC interventions on quality of life	Not observed (compared to the significant effect in men)
**As a patient**	**Sex-related aspects of doctor-patient communication**
Perceiving of symptom burden	Female patient has to report higher level of distress in order to get their symptoms acknowledged, documented and managed
**As informal caregiver of a person with advanced HF**	**Women compared to Men**
Burden and quality of life	Greater degree of mental and physical strain, higher level of distress and burdenWorse QoL
Social expectations	More often related to the role of women as caregivers for family member at the end-of-life
Psychological distress	More related to spouses' health condition (more distress in healthy wives of patients than healthy husbands of patients)

## Conclusions

People living with HF are confronted with suffering caused by physical, emotional, existential and spiritual problems despite optimal cardiologic care, usually during the long journey of living with this syndrome, and not only at the end-of-life. Symptom management requires close cooperation between cardiology and other disciplines including PC. Implementing PC for all those with health-related needs as soon as they emerge could improve their QoL. PC is underused and offered to the minority of people living with HF in the very last moments of their life. Putting suffering in the center of care requires clinicians to attend to the individual experiences of persons' illness, to address its physical, psychological, spiritual and social burdens, and to support the patient in the journey to real healing by careful listening and witnessing. However, this very individual approach should not be the reason for ignoring the impact of different factors such as sex on how those individuals usually or more often experience illness, how they react to treatment, or cope with the suffering. Data suggest that PC interventions need to be more specific to women vs. men. This specificity may involve sex related symptoms prevalence and intensity, efficacy of symptom management, response to pharmacotherapy, identifying comorbidity and additional symptoms related to it, specific social challenges such as widower status or loneliness, up to different spiritual coping and needs. The differences between both sexes really matter in the way people perceive their life, its quality and the support they receive, and they should be acknowledged when providing medical care.

## Author Contributions

All authors contributed to designing the scope of the paper, have written parts of the text, reviewed, and adjusted the whole manuscript.

## Conflict of Interest

The authors declare that the research was conducted in the absence of any commercial or financial relationships that could be construed as a potential conflict of interest.
